# Primary breast lymphoma of childhood: a case report and review of literature

**DOI:** 10.1186/s12887-021-03002-6

**Published:** 2021-11-30

**Authors:** Giulia A. Restivo, Marta Pillon, Lara Mussolin, Clara Mosa, Angela Guarina, Angela Trizzino, Salvatore Ialuna, Elisa Carraro, Emanuele S.G. D’Amore, Giovanna Russo, Caterina Elia, Maurizio Mascarin, Adriana Zangara, Paolo D’Angelo, Piero Farruggia

**Affiliations:** 1 Department of Health Promotion, Mother and Child Care, Internal Medicine and Medical Specialties “G. D’Alessandro”, University of Palermo, Palermo, Italy; 2 Clinic of Pediatric Hemato-Oncology, Department of Women’s and Children’s Health, University of Padova, Padova, Italy; 3 Institute of Pediatric Research - Fondazione Città della Speranza, Padova, Italy; 4 Department of Pediatric Hemato-Oncology, ARNAS Ospedali Civico, G. Di Cristina, Palermo, Italy; 5 Nuclear Medicine Unit, Ospedali Riuniti Villa Sofia-Cervello, Palermo, Italy; 6 Department of Pathology, San Bortolo Hospital, Vicenza, Italy; 7 Pediatric Hematology and Oncology Unit - AOU Policlinico “Rodolico-San Marco”, University of Catania, Catania, Italy; 8 AYA Oncology and Pediatric Radiotherapy Unit, CRO - Centro di Riferimento Oncologico di Aviano, IRCCS, Aviano, Italy; 9 Radiology Unit, Radiology Department, A.R.N.A.S. Ospedali Civico, Di Cristina e Benfratelli, Palermo, Italy

**Keywords:** Primary breast lymphoma, rituximab, Children

## Abstract

**Background:**

Primary breast lymphoma (PBL) is an extremely rare neoplasm in children; by definition, it manifests in the breast without evidence of lymphoma elsewhere, except ipsilateral axillary nodes.

**Case presentation:**

We report a case of a 15-year-old girl diagnosed with diffuse large B-cell lymphoma (DLBCL) of the right breast: the patient received chemotherapy and rituximab, achieving complete remission. A literature review revealed other 11 cases of pediatric PBL; it mainly affects female adolescents and can involve right and left breast equally. Different histologic subtypes have been described, arising from both B-cell and T-cell. Therapeutic approaches were very different, from chemotherapy to local treatment with surgery and/or radiotherapy.

**Conclusions:**

Our case is the first in which rituximab was administered, suggesting to be a promising therapy in B-cell PBL, as already demonstrated in pediatric B-cell lymphoma from other sites. Further investigations are needed to identify prognostic factors and establish the most effective treatment.

## Background

Breast malignancy is a rare disease in childhood; it can be a primary or a secondary neoplasm. The latter is the most common: in this case, the breast is involved as a metastatic site of different cancer originated elsewhere, such as rhabdomyosarcoma, neuroblastoma, lymphoma, or leukemia. Primary tumors, instead, arise from the breast, and in childhood, there are occasional reports about carcinoma [1], rhabdomyosarcoma [2], and lymphoma [3].

Primary breast lymphoma (PBL) is an extremely rare non-Hodgkin lymphoma (NHL); it represents less than 3% of all extranodal NHL and its onset in childhood is exceptional [4]. The criteria that must be fulfilled for the PBL diagnosis were defined by Wiseman and Liao in 1972 [3]: (i) adequate pathological specimen with evidence of both mammary tissue and lymphomatous infiltrate, (ii) absence of concurrent metastatic disease (only the involvement of homolateral axillary nodes is acceptable), (iii) absence of a previous diagnosis of extramammary lymphoma.

We report a case of PBL in a 15-year-old girl, who was treated with chemotherapy plus rituximab. A review of the pediatric literature on this topic was conducted thereafter.

## Case presentation

**Fig. 1:**
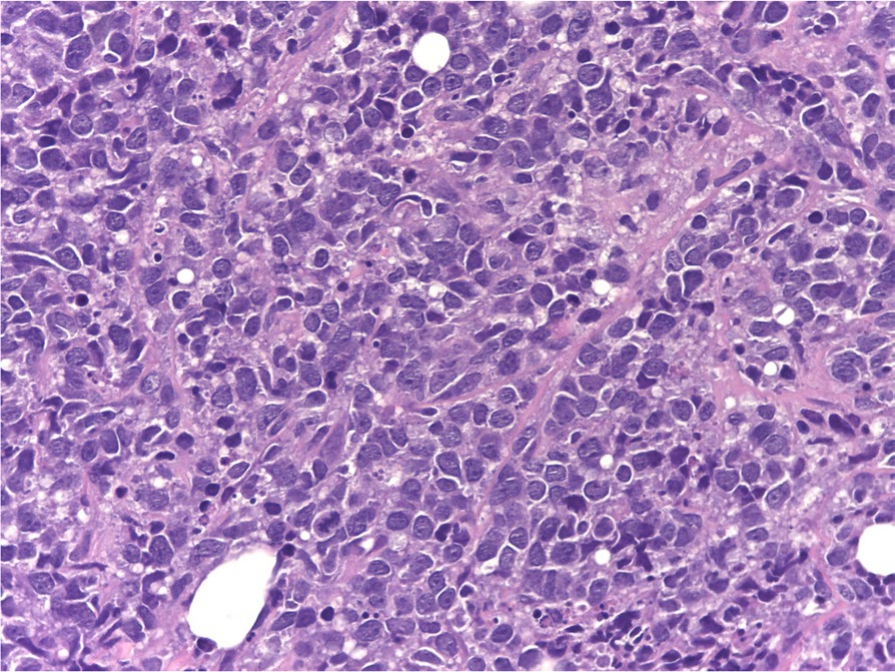
Hematoxylin-eosin staining

**Fig. 2:**
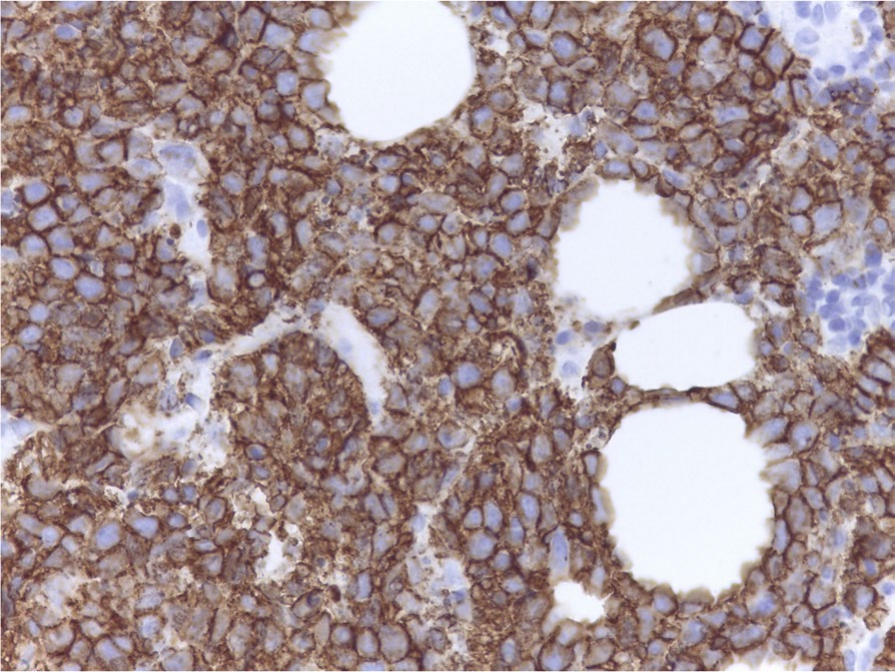
CD20 staining

**Fig. 3:**
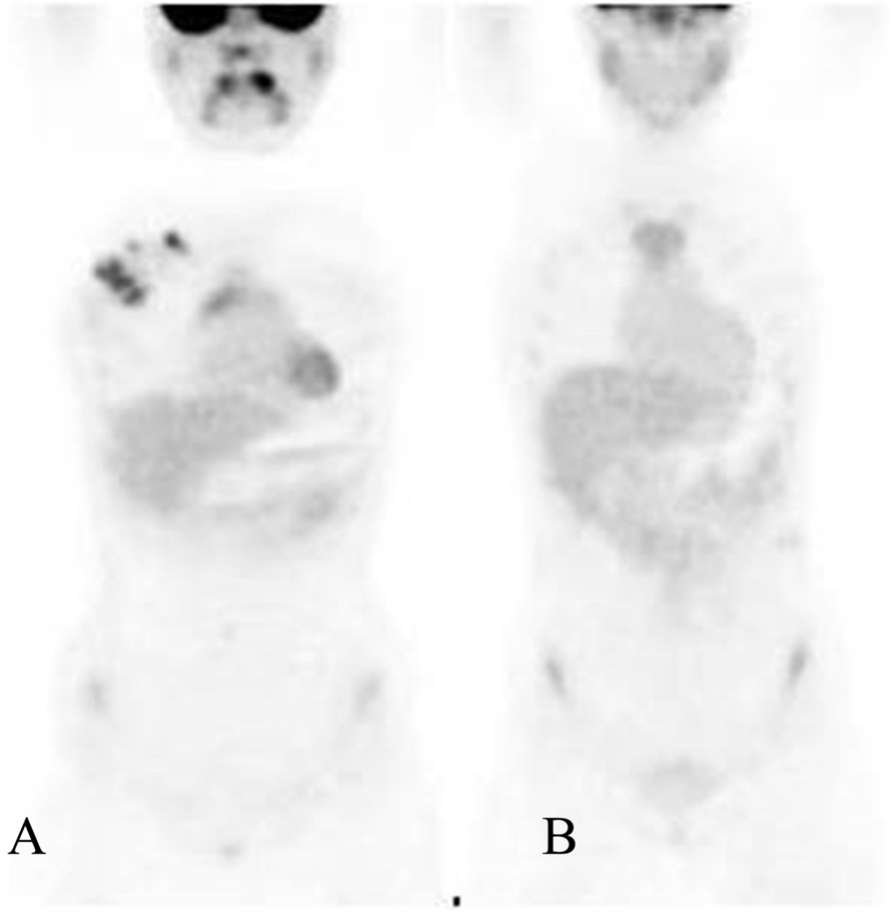
Whole body PET-CT scan: pre- (A) and post- (B) treatment

A 15-year-old girl presented with a palpable mass in her right breast, which she has had for 2 months; during this time, she noticed that the lump had not increased in size. She did not have systemic symptoms or a history of weight loss. During the physical examination, two solid and painless masses were appreciated in the upper-outer and the central-low quadrants; the skin overlying the lesions was completely normal and there were no palpable lymph nodes. Blood count, liver and kidney function tests, erythrocyte sedimentation rate (ESR), lactate dehydrogenase (LDH), and uric acid blood levels were in the normal range. Ultrasound (US) showed a 6 cm hypervascular mass with indistinct margins in the upper-outer quadrant and other two smaller masses (diameter 1.9 cm and 2.1 cm, respectively), probably connected to the largest one, in the central-low portion; moreover, two enlarged homolateral axillary lymph nodes (maximal diameter 1.7 cm) were identified. On breast magnetic resonance imaging (MRI), a 6.5 cm single and irregular mass with intense contrast enhancement and three suspected axillary nodes (maximal diameter 1.5 cm) were described. US-guided needle biopsy of the right breast lesion was performed; analysis was carried out with a Leica microscope (dm2000 led, Objective: x63), Leica ICC50HD camera and Leica Acquire software for Macbook pro 16’ 2,6 GHz 6-Core Intel Core i7; histologic examination (Figs. 1 and 2) showed a diffuse large B-cell lymphoma, not otherwise specified (DLBCL, nos), CD19+, CD20+, of germinal center origin according to immunohistochemical algorithms (CD10+, BCL6+, IRF4/MUM1+, FoxP1+, LMO2+, HGAL+), with high proliferation index (MIB-1 80%). A high expression of c-MYC protein was found (70%); however, fluorescence in situ hybridization (FISH) analysis was negative for MYC translocation (Vysis break apart and IGH/MYC dual fusion translocation probes) as well as for BCL2 and BCL6 translocations (Break apart translocation probes), excluding a high-grade B-cell lymphoma with double hit. Finally, no evidence of Epstein-Barr virus (EBV) was found with in situ hybridization for the Epstein-Barr virus-encoded small RNAs (EBERs). The staging was completed with bone marrow aspirate, lumbar puncture, brain MRI, neck-thorax-abdomen computerized tomography (CT) scan, and whole-body positron emission tomography (PET)-CT scan (Fig. 3 A); a high fluorodeoxyglucose (FDG) uptake with a maximum standardized uptake value (SUVmax) of 11.5 was evidenced in the right breast. No other organ involvement was ascertained. According to St. Jude staging [5] and the new revised International Pediatric Non-Hodgkin Lymphoma Staging System (IPNHLSS) [6], the stage was IIE (single extranodal tumor with regional node involvement). The girl received intensive combination chemotherapy according to the AIEOP LNH-97 trial (risk group 3) [7] plus rituximab (Table 1). After the second block of chemotherapy, breast MRI and whole-body PET-CT scan showed a complete radiologic and metabolic response (Fig. 3B). These examinations were repeated at the end of the treatment, confirming the disappearance of any lesions. The total duration of chemotherapy was approximately 3 months and the girl is alive without any evidence of disease after 20 months from the end of treatment.

**Table 1:** Therapy courses according to AIEOP LNH-97 protocol plus rituximab. According to the treatment risk group (R3), patients received the following chemotherapy cycles: prephase, AA, BB, CC, AA, BB

		Days					
_**Drug**_	Dose	0	+1	+2	+3	+4	+5
_**Prephase**_							
_Dexamethasone orally/IV_^a^	_mg/sqm_		_5_	_5_	_10_	_10_	_10_
_Cyclophosphamide IV (1 h)_	_200 mg/sqm/day_		_x_	_x_			
_MTX+ARA-C+PDN IT_	_12 mg +30mg+10mg_^b^		_x_				
_**Cycle AA**_							
_*Rituximab IV*_	_*375 mg/sqm*_	_*x*_^f^					
_Dexamethasone orally/IV_^a^	_10 mg/sqm_		_x_	_x_	_x_	_x_	_x_
_Vincristine IV_^c^	_1.5 mg/sqm_		_x_				
_Methotrexate IV_^d^	_5 g/sqm_		_x_				
_Ifosfamide IV (1 h)_	_800 mg/sqm_		_x_	_x_	_x_	_x_	_x_
_Etoposide IV (2 h)_	_100 mg/sqm_					_x_	_x_
_Cytarabine IV (1 h)_	_150 mg/sqm_					_x -x_^e^	_x - x_^e^
_MTX+ARA-C+PDN IT_	_12 mg +30mg+10mg_^b^		_x_				
_**Cycle BB**_							
_*Rituximab IV*_	_*375 mg/sqm*_	_*x*_					
_Dexamethasone orally/IV_^a^	_10 mg/sqm_		_x_	_x_	_x_	_x_	_x_
_Vincristine IV_^c^	_1.5 mg/sqm_		_x_				
_Methotrexate IV_^d^	_5 g/sqm_		_x_				
_Cyclophosphamide IV (1 h)_	_200 mg/sqm/day_		_x_	_x_	_x_	_x_	_x_
_Doxorubicin IV (4 h)_	_25 mg/sqm_					_x_	_x_
_MTX+ARA-C+PDN IT_	_12 mg +30mg+10mg_^b^		_x_				
_**Cycle CC**_							
_*Rituximab IV*_	_*375 mg/sqm*_	_*x*_					
_Dexamethasone orally/IV_^a^	_20 mg/sqm_		_x_	_x_	_x_	_x_	_x_
_Vindesine IV_^c^	_3 mg/sqm_		_x_				
_Cytarabine IV (3 h)_	_3 g/sqm_		_x -x_^e^	_x - x_^e^			
_Etoposide IV (2 h)_	_100 mg/sqm_					_x_	_x_
_Mtx+ARA-C+PDN IT_	_12 mg +30mg+10mg_^b^		_x_				

Abbreviations: MTX: methotrexate; ARA-C: cytarabine; PDN: prednisolone; IV: intravenously; h: hours; IT: intrathecal; CNS: central nervous system; sqm: square meters

^a^subdivided in 3 doses

^b^Dose of IT chemotherapy was age-adjusted for children less than 3 years. In courses AA and BB, IT therapy was administered 2 h after beginning of MTX IV

^c^Maximum dose was 2 mg

^d^10% of MTX dose was given in 0.5 h, 90% of dose over 23.5 h. L-leucovorin rescue IV was 15 mg/sqm at h 42, 7.5 mg/sqm at h 48, and 54 after beginning of MTX

^e^Doses were 12 h apart

^f^For the first course AA, rituximab infusion on day 0 corresponded to day 5 of prephase

## Discussion and conclusion

PBL is a rare form of extranodal lymphoma, affecting females in almost all cases. It is much more common in adulthood where it is difficult to distinguish clinically from breast carcinoma because both neoplasms typically present with a painless breast mass; in adults, the right breast is more involved than the left one and the most common histology is DLBCL [4]. A rare histologic type, the anaplastic large cell lymphoma (ALCL), predominates in women with breast implants [8].

PBL is an exceptional breast malignancy in the pediatric age; in literature, there are 11 case reports on this topic (Table 2) [9–19]. Although in other studies [20–25] some PBL patients aged from 15 years were reported, it was not possible to include these children in our review because no detailed information was presented. From the analysis of these 12 well-described pediatric patients (Table 3), it can be deduced that PBL mainly affects female adolescents (median and mean age 14.5 and 14.2 respectively) with only one 11-year-old boy; as in adulthood, there is a slight predominance of the right breast (6 out of 10 patients for whom the data are available), with only 1 girl showing bilateral involvement [15].

**Table 2:** Main features of pediatric PBL patients reported in the literature and our case

Reference	Age/Sex	Side	Size (cm)	Stage	Histology	Treatment	Outcome (from onset)
Dixon *et al.* 1987 [9]	17y/F	NA	2	NA	NHL unclassified	Surgery + RT	CR, 25 years
Boothroyd *et al.* 1994 [10]	11y/F	Right	NA	NA	B-cell NHL	CT	CR, 36 months
Rogers *et al.* 1994 [11]	14y/F	NA	8 x 7	IIE	LBL	CT	CR
Aguilera *et al.* 2000 [12]	13y/F	Left	6 x 6	IE	ALCL ALK +	Surgery	Dead
Barista *et al.* 2000 [13]	17y/F	Right	NA	IIE	DLBCL	CHOP + RT	CR, 57 months
Abdullah *et al.* 2004 [14]	15y/F	Left	5	IE	ALCL ALK +	CT (cyclophosphamide, prednisolone)	CR, 22 years
Lingohr *et al.* 2009 [15]	12y/F	Bilateral	NA	NA	BL	Surgery	Dead
Daneshbod *et al.* 2010 [16]	16y/F	Right	NA	IIE	ALCL ALK +	CHOP	Dead
Ishizuka *et al.* 2020 [17]	14y/F	Right	4	IE	LBL	CT (ALL, not specified trial)	CR, 8 months
Charfi *et al.* 2020 [18]	16y/F	Left	NA	IIE	ALCL ALK +	CT (doxorubicin, bleomycin, vincristine, dacarbazine)	CR, 24 months
Oral *et al.* 2021 [19]	11y/M	Right	3.5 x 1.7	IE	LBL	Surgery	NA
Our case	15y/F	Right	6.5	IIE	DLBCL	CT (AIEOP LNH-97 trial)	CR, 20 months

Abbreviations: years; NA: not available; RT: radiotherapy; CR: complete remission; LBL: lymphoblastic lymphoma; CT: chemotherapy; ALCL: anaplastic large cell lymphoma; ALK: anaplastic lymphoma kinase; DLBCL; diffuse large B-cell lymphoma; CHOP: cyclophosphamide + doxorubicin + vincristine + prednisone; BL: Burkitt lymphoma; ALL: acute lymphoblastic leukemia

**Table 3:** Main features of pediatric PBL patients sorted by frequency

N°	12	Frequency (%)
**Age (years)**		
Median/Mean	14.5/14.2	-
**Sex**		
Female	11	91.7
Male	1	8.3
**Side**		
Unilateral	9	75
Bilateral	1	8.3
Unknown	2	16.7
**Size**		
≤ 5 cm	4	33.3
> 5 cm	3	25
Unknown	5	41.7
**Histology**		
ALCL	4	33.3
LBL	3	25
DLBCL	2	16.7
BL	1	8.3
B-cell NHL	1	8.3
NHL unclassified	1	8.3
**Stage**		
IE	4	33.3
IIE	5	41.7
Unknown	3	25
**Local treatment**		
Surgery only	3	25
Surgery + RT	1	8.3
**Chemotherapy**		
CT only	7	58.3
CT + RT	1	8.3
**Outcome**		
CR	8	66.6
Dead	3	25
Unknown	1	8.3

Abbreviations: ALCL: anaplastic large cell lymphoma; DLBCL: diffuse large B-cell lymphoma; BL: Burkitt lymphoma; LBL: lymphoblastic lymphoma; LNH: non-Hodgkin lymphoma; CT: chemotherapy; RT: radiotherapy; CR: complete remission

US with color-Doppler is frequently the first imaging technique, being not invasive and able to distinguish benign from malignant breast lesions in the majority of cases; on US, malignancies are typically hypoechoic masses with indistinct margins, irregular shape, and intense vascularization [26]. On MRI, PBL is characterized by hypointensity or isointensity at T1-weighted imaging and by hyperintensity at T2-weighted imaging, with homogenous or heterogeneous enhancement [27]. PET-CT, highly sensitive and specific in the tumor staging and the evaluation of treatment response, can show unifocal, multifocal, or diffuse high FDG uptake [28]. In agreement with the criteria proposed by Wiseman and Liao [3], PBL without axillary nodes involvement is classified as stage IE and, in case of loco-regional node metastases, as stage IIE; among the 9 patients in whom the stage is reported, there are 4 stage IE and 5 stage IIE. Histologic subtypes are available in 11 patients: 4 ALCL anaplastic lymphoma kinase (ALK) positive, 3 lymphoblastic lymphomas, 1 Burkitt lymphoma, 2 DLBCL, and 1 B-cell NHL not specified. This seems to be in discordance with adulthood where DLCBL is the predominant subtype (about 70% of all patients) [23].

As regards the treatment, different therapeutic approaches were used: 7 received chemotherapy, 3 patients underwent breast surgery only, and in 2 patients radiotherapy was added to surgery or chemotherapy. Currently, surgery is not considered a therapeutic choice. A large meta-analysis on patients with PBL aged from 17 to 95 years [23] showed that mastectomy offers no benefit in terms of event-free survival (EFS) and overall survival (OS). The current standard therapy for children affected by Burkitt lymphoma or DLBCL consists of chemotherapy tailored according to stage, LDH value, and disease dissemination; furthermore, the outcome of these patients has been recently improved by the addition of rituximab [29, 30], an anti-CD20 monoclonal antibody already used in the treatment of adult primary breast DLBCL [31]. Our girl is the first PBL pediatric patient to be treated with polychemotherapy plus rituximab, probably because most of the patients analyzed in the present review were in the pre-rituximab era.

In our review, 8 children showed complete response to treatment, 3 girls (2 with ALCL) died for progression of disease, and in 1 patient the outcome was not specified; none of the analyzed clinical data, such as tumor size, unilateral or bilateral involvement, and disease stage seems to be associated with an unfavorable outcome. In adult patients, controversial results have been found: in Wong et al. [25], who evaluated 26 adult patients with primary breast NHL, the only significant prognostic factor for survival was the Ann Arbor stage; according to Hu et al. [22], who collected data on 108 patients with primary breast DLBCL aged between 16 and 85 years, tumor size larger than 5 cm and regional node involvement were not associated with significant changes in EFS or OS, while according to the meta-analysis of Jennings et al. [23] on 465 patients with PBL (mean age 54 years), nodal status was the best predictor of survival. Furthermore, it has not yet been investigated whether the different histological subtypes are correlated to prognosis.

In conclusion, PBL is a rare and poorly investigated type of NHL in childhood, with most of the information resulting from studies performed on adults. In pediatric patients, it mainly affects female adolescents and the most common presentation is a unilateral breast mass. Different lymphoma subtypes have been described and, unlike adult patients, DLBCL seems not to be the most frequent histology. No prognostic factors have been clearly determined and different therapeutic approaches have been used, from chemotherapy to local treatment with surgery and/or radiotherapy. Our case is unique because it is the first pediatric primary breast DLBCL patient treated with chemotherapy plus rituximab. Further pediatric researches are needed in order not only to verify the efficacy of rituximab and improve treatment strategies but also to identify specific molecular features and prognostic factors.

## Data Availability

The data and materials of this case report are available from the corresponding author upon reasonable request.
